# von Hippel-Lindau Syndrome: Genetic Study of Case With a Rare Pathogenic Variant With Optic Nerve Hemangioblastoma, a Rare Phenotypic Expression

**DOI:** 10.3389/fonc.2020.00139

**Published:** 2020-02-14

**Authors:** Sandra Di Felice Boratto, Pedro Augusto Soffner Cardoso, Denise Gonçalves Priolli, Ricardo Vieira Botelho, Alberto Goldenberg, Bianca Bianco, Jaques Waisberg

**Affiliations:** ^1^Department of Surgery, Faculdade De Medicina Do ABC, Santo André, Brazil; ^2^Department of Surgery, State Public Servant Hospital (IAMSPE), São Paulo, Brazil; ^3^Postgraduate Programme Stricto Sensu in Health Science, Sao Francisco University Medical School, Bragança Paulista, Brazil; ^4^Department of Surgery, Escola Paulista de Medicina, São Paulo Federal University, São Paulo, Brazil; ^5^Department of Collective Health, Faculdade De Medicina Do ABC, Santo André, Brazil

**Keywords:** von Hippel-Lindau syndrome, hemangioblastoma, optic nerve neoplasms, brain neoplasms, renal carcinoma, *VHL* gene

## Abstract

von Hippel-Lindau syndrome (VHLS) is a rare, autosomal dominant genetic disease with high penetrance and variable phenotypic expression caused by variants in the *VHL* gene. VHLS is associated with the presence of vascular tumors, often hemangioblastoma of the central nervous system, retina, or spinal cord and, less frequently, pancreatic cystic neoplasm, pancreatic neuroendocrine tumor, clear cell carcinoma of the kidney, endolymphatic sac tumor, pheochromocytoma, and paraganglioma. The authors report a case of a patient with VHLS with a rare pathogenic variant in the *VHL* gene and with an optic nerve hemangioblastoma, a rare phenotypic expression.

**Case report:** A 49-year-old woman was diagnosed with cystic neoplasm of the pancreas, renal cell carcinoma of the right kidney, and hemangioblastoma of the left optic nerve. The patient's family history revealed siblings with VHLS manifestations. The index case was her mother who died at age 63 of clear cell renal carcinoma. The information was obtained by consulting the patient's medical register and by interviews with the patient and her relatives. The presence of left optic nerve hemangioblastoma was suggested by CT scan of the skull and orbit. The sequencing of the *VHL* gene was performed in the peripheral blood by the polymerase chain reaction (PCR) technique, and the duplication and deletion research was performed using the multiplex ligation-dependent probe amplification (MPLA) technique. The presence of a rare pathogenic variant c.263G> A (p.Trp88Ter) was observed in heterozygosity in the *VHL* gene that determined a premature stop codon. CT scan of the skull and orbits suggested the presence of HB in the optic nerve of the left eye. The results of the CT scan of the skull and orbits show thickening with tortuosity of the left optic nerve, with a small area of nodular enhancement. The right optic nerve had a conserved aspect.

**Conclusion:** This is the fourth case described of this rare pathogenic variant of the *VHL* gene, according to the Human Gene Mutation Database and VHLdb database records and with an optic nerve hemangioblastoma of the optic nerve, a very rare phenotypic expression of the VHLS.

## Introduction

The von Hippel-Lindau syndrome (VHLS), also known as familial cerebello retinal angiomatosis, is an autosomal dominant genetic disease with high penetrance and variable expressivity (1). VHLS is characterized by the presence of benign and/or malignant tumors, most of them of vascular origin, with the most frequent being hemangioblastoma (HB), usually located in the central nervous system (CNS), mainly in the brain and cerebellum (1, 2), but also founded in the spinal cord and retina (1). Less than 5% of HBs present in the supratentorial compartment and optic nerve HBs are extremely rare, with the vast majority of this tumor occurs in patients with VHLS (3, 4).

Other non-vascular benign and malignant tumors that may form part of SVH are pancreatic cystic neoplasm, pancreatic neuroendocrine tumor, clear cell renal carcinoma (CCR), endolymphatic sac tumor, pheochromocytoma (PCC), paraganglioma (1, 2, 5), endolymphatic sac tumor, and papillary cystadenoma of the epididymis (1, 2, 5).

VHLS is determined by the presence of germline or somatic pathogenic variants in the *VHL* gene (von Hippel-Lindau tumor suppressor, Gene ID7428), mapped at 3p25.26 (1, 2).

The incidence of VHLS ranges from 1 case in every 36,000 to 45,000 live births (6, 7). The natural history of the disease, including the manifestation of tumors and their severity, is highly variable among affected families, even cases within the same family (1, 2, 5, 6). The mean age of the first manifestation is 26 years, although it can affect patients from 1 to 70 years (1, 2, 5). The disease is highly penetrating, and 90% of patients develop symptoms before age 65 (1). An occurrence of genetic anticipation has been observed that determines the involvement of patients at progressively earlier ages and with more severe manifestations in successive generations (2).

Usually, the initial clinical manifestations in 60 to 80% of the patients with VHLS are due to the presence of HB in the CNS (1). Despite the benign nature of these tumors, they represent the major cause of morbidity and mortality due to compression exerted by their growth or by spontaneous bleeding of the vascular component of these tumors in the CNS structures (1, 2, 5).

The aims of this study were to describe a patient with VHLS with a rare *VHL* pathogenic variant and a rare phenotypic manifestation represented by HB of the left optic nerve. To the best of our knowledge, this pathogenic variant was described just in other three cases reported, and until now, 20 cases of optic nerve HB have been reported with 14 of them associated with VHLS (3).

## Case Presentation

### Clinical Report

**Figure 1:**
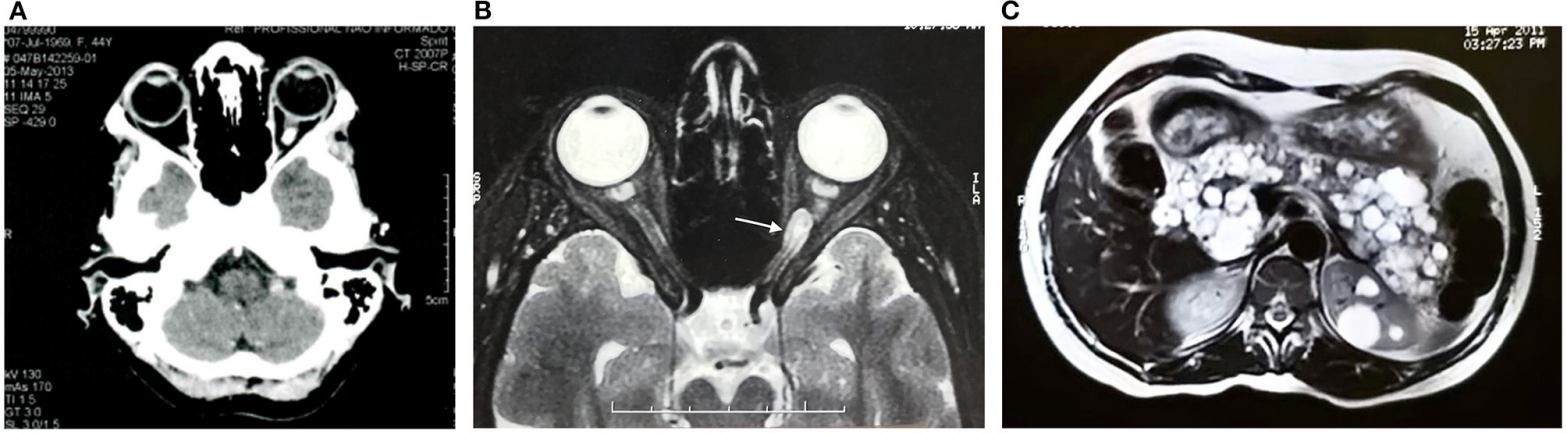
Computed tomography **(A)** and magnetic resonance imaging of the skull and orbit **(B)**, and computed tomography of the abdomen **(C)** of the von Hippel- Lindau syndrome proband. **(A)** Left optic nerve thickening with tortuosity and an enhancement area with nodular aspect. **(B)** Left intraorbital tumor on T1-weighted imaging (white arrow). **(C)** Pancreatic cysts and renal cysts.

**Figure 2:**
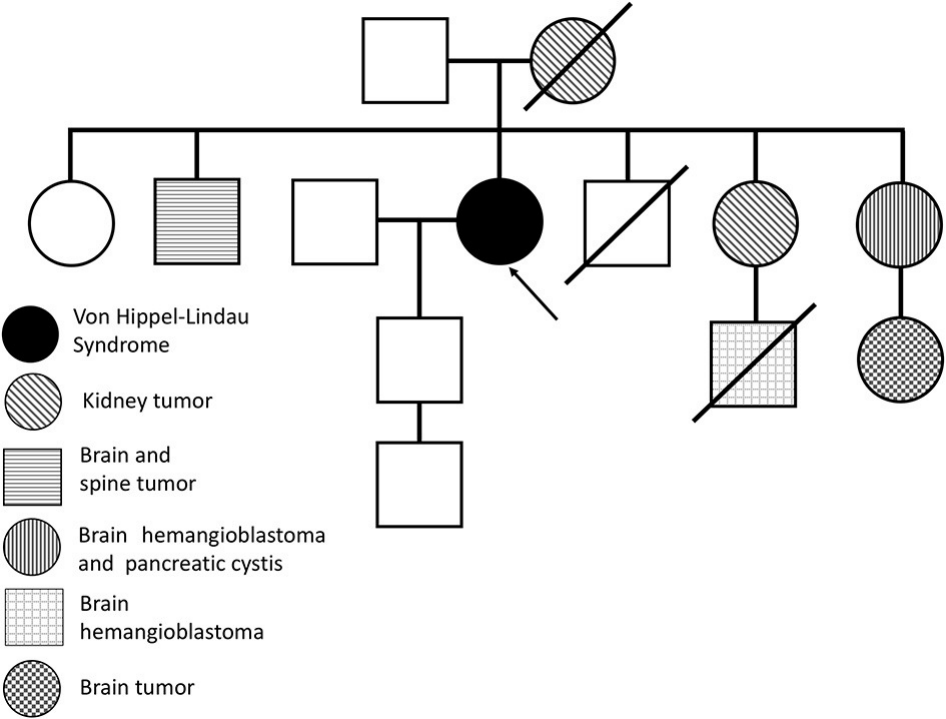
Familiar pedigree of the proband (arrow) with von Hippel-Lindau syndrome.

Our case reporting followed CARE recommendations (8). A 49-year-old woman had sporadic episodes of rotational vertigo 12 years ago and abdominal pain 10 years ago. Ultrasonography and magnetic resonance imaging (MRI) of the abdomen revealed the presence of pancreatic cysts that did not show significant growth in imaging tests. Seven years ago, a computed tomography scan (CT) of the abdomen showed a solid mass in the lower pole of the right kidney. The patient was then submitted to right partial nephrectomy, and pathological examination revealed the presence of CCR. There was no need for adjuvant treatment. In the same year, the patient complained of altered visual acuity and a CT scan of the skull and orbit was made. The results show thickening with tortuosity of the left optic nerve, with a small area of nodular enhancement, with an extension about 0.8 × 0.5 × 0.4 cm. The right optic nerve had a conserved aspect. These results were considered suggestive of the presence of HB in the optic nerve of the left eye (Figure 1A). MRI of the head showed a gadolinium-enhancing well-circumscribed mass on the left optic nerve in the orbital (Figure 1B). The mother of the patient died at age 63 from an advanced kidney tumor, and her father is alive and healthy at age 80. The patient has one child, currently 27 years old, who has a recurrent headache complaint but refuses to undergo the investigation for VHLS, and a healthy, symptom-free, five-year-old grandson. The patient has five brothers and two sisters. One sister underwent partial nephrectomy due to carcinoma in the right kidney without the need for adjuvant treatment, another sister was operated on by HB from the CNS and had pancreatic cysts, and one brother had two HBs removed that were located in the brain and spine. A nephew of the patient at age 12 was diagnosed with HB from the CNS and died 4 years later due to complications of brain tumor that did not come to be removed. Another niece was diagnosed at age 21 of cerebral HB, and 6 months ago the tumor had resected. The family pedigree of the proband is shown in Figure 2. To date, the patient has no manifestation of PCC, is under urological and endocrinological follow-up, and is submitted to abdominal CT every year and to orbit and CNS MRI every two years. In the control examinations, the patient maintains images of pancreatic and renal cysts (Figure 1C) without images of renal tumor recurrence or metastases. The right optic nerve is intact, and there are no CNS lesions in the MRI of the brain. She has only a slight decreased of the visual acuity in the left eye due to the optic nerve HB and does ophthalmologic follow up each 2 years.

## Methods

This study was performed according to the guidelines of the Declaration of Helsinki, fulfilling all requirements for retrospective studies in humans. Clinical data, family data, images and peripheral blood sample were collected only after signing of the Informed Consent Terms approved by the Institutional Research Ethics Committee.

### Sequencing Sanger Analysis

One mL of peripheral blood was collected on filter paper to extract genomic DNA (Qiagen, Germantown, MD, USA). The *VHL* gene sequence (OMIM: 608537) was amplified by polymerase chain reaction (PCR), and sequencing was performed from both the DNA strands of the entire coding region and the intron and junction regions. The product of the sequencing was compared to a reference sequence of GRCh37 human genome (9).

## Results

Sequencing of the *VHL* gene revealed a pathogenic variant c.263G>A [(VHL:Ch3(GRCh37):g.10183794G>A, NM_000551.3:c.263G>A, p.Trp88Ter] in heterozygosis, as confirmed by the Human Gene Mutation Database (HGMD®) (10). This variant has previously been described as causing VHLS by Gallou et al. and by Wong et al. According to the American College of Medical Genetics and Genomics (11) this variant is classified as pathogenic (clinical test, variant ID: 182978) and Class 1.

## Discussion

The present study reports the case of a woman with VHLS who manifested clinically due to the presence of pancreatic cystic neoplasia, RCC in the right kidney, and HB in the optic nerve of the left eye. The diagnosis of VHLS was confirmed by the identification of the pathogenic variant *VHL:*c.263G>A in heterozygosis. According to the Human Gene Mutation Database (10) and VHLdb (12) databases, only three other cases have been described until now with the same pathogenic variant (13–15) (Table 1).

**Table 1:** Published reports of patients with von Hippel-Lindau syndrome with pathogenic variant c.263G> A (p.Trp88 *) described in the Human Gene Mutation Database^10^.

**References**	**Type of genetic variant**	**Phenotype**	**Streaming**
Mattocks et al. (15)	TS/NS	Not described	Germinative
Gallou et al. (13)	TS/NS	CCR	Somatic
Gallou et al. (14)	TS/NS	CCR	Somatic
Present report	NS	CCR, optic nerve HB, cystic pancreatic neoplasm	Germinative

*TS, transition; NS, nonsense; CCR, renal cell carcinoma; HB, hemangioblastoma*.

In the patient of this report, *VHL* gene sequencing identified a pathogenic variant that determined a premature stop codon that predisposes to exuberant clinical manifestation. The inheritance of this *VHL* variant, according to the proband's heredogram, is possibly maternal, since the mother of the proband died as a consequence of renal cancer complications.

The *VHL* gene has four exons, but only three of them are coding (exon 1: codon 1 to 113; exon 2: codon 114 to 154; and exon 3: codon 155 to 213) (16). These exons can be transcribed alternately from codons 1 and 54. The gene products are two isoforms of the VHL protein, 1>30-kDa (VHL30) composed of 213 amino acids and a smaller one (VHL19) of 19-kDa, which results in a protein of 160 amino acids translated from codon 54 (17, 18).

Additional pathogenic variants for CCRs were found (18), indicating that VHLS-associated neoplasms may not be caused exclusively by pathogenic variants in the *VHL* gene but also by pathogenic variants in the hypoxia-inducible factor 1-alpha (*HIF-1A*) gene or in other genes, such as *PBRM1, SETD2*, and *BAP1*(19–23). The VHL protein participates in the induction and degradation of the HIF-1A protein and in the presence of an anomalous VHL protein, the involvement of VHL in the degradation of HIF-1A is inhibited and proteasomal cleavage is suppressed (23–25). The entry of HIF-1A into the nucleus promotes the transcriptional activation of pro-angiogenic genes, such as vascular endothelial growth factor (*VEGF*), platelet-derived growth factor beta (*PDGF-*β), transforming growth factor alpha (*TGF-*α), chemokine receptor type (*CXCR4*), erythropoietin (*EPO*), and glucose transport 1 (*GLUT-1*) (25), leading to vascular proliferation and angiogenesis, influencing extracellular matrix remodeling, and increasing resistance to apoptosis (26–29). Uncontrolled angiogenesis contributes to the hypervascular nature of tumors that develop in VHLS (25, 27).

*VHL* is ubiquitously expressed in 27 different tissues of the human organism (30), and the mutated gene may also result in abnormal regulation of the *TP53* gene, the NF-kB (kappa-light-chain-enhancer of activated B cells) pathway, retinol-binding protein, remodeling factor of p400 chromatin, extracellular matrix, and cytoskeleton (31), events that may contribute to the formation of tumors observed in the VHLS, as occurred in the patient of this report.

Several germline pathogenic variants were identified in the *VHL*, the most frequent being the missense variants that occurs in 27 to 52% of the cases, followed by the non-sense variants that is present in 13 to 27% of the cases (31, 32).

However, a broad spectrum of pathogenic variant types have been described, including frameshift, large deletions or microdeletions, gene rearrangements, deletions or inframe insertions, and, more rarely, variants at splicing sites (32). Somatic inactivation of the wild-type *VHL* allele may occur due to allelic loss, hypermethylation, or point mutation (1). According to the Universal Database (33), there are 1,230 pathogenic variants described in the *VHL* gene, among which 55.8% are missense and 9.9% are non-sense.

In addition, deletions of the *VHL* gene in exons 1, 2, and 3 involving the neighboring gene *C3orf10* or *BRK1* (SCAR/WAVE actin nucleating complex subunit) that participates in the actin nucleation process may decrease the rate of cell growth and lead to a lower incidence of CCR in patients with VHLS (1).

An analysis of pathogenic variants in 945 Dutch families with VHLS found that type 1 had 43% of missense variants, 17% of frameshift, 13% of non-sense, 9% of splicing, 8% insertion/deletion inframe, and 10% partial/complete deletions (32). VHLS type 2 was associated with missense variants that compromised VHL protein binding sites and was found in 83.5% of families (32). Patients with a complete deletion of the *VHL* gene have a decreased incidence of HB in comparison with patients with missense variants (3). The patient of the present report has a non-sense variant in heterozygosity that determined a premature stop codon (p.Trp88^*^). Individuals who meet VHLS criteria with multiple organ involvement carry identifiable *VHL* pathogenic variants in almost 100% of cases (18).

In VHLS, the growth pattern of HB may be linear, exponential, and/or fluctuating and there may be more than one tumor with different growth patterns between them in the same patient (7). Therefore, HBs in VHLS have a worse prognosis than those found in the general population (5). In the CNS, HB can be found in the cerebellum in 16–69% of cases, brainstem in 5–22%, spinal cord in 13–53%, equine tail in 11%, and supratentorial region in 1–7% of patients with VHLS (1).

Multiple bilateral renal cysts are found in 50–70% of patients (7). In general, renal cysts are asymptomatic and rarely result in renal failure, even when multiple and bilateral (7). Multiple and bilateral RCCs occur, respectively, in 30–45% and 30–50% of symptomatic patients who already have metastases, and lymph node metastases are described in 15% of CCR cases (7, 34). Hematogenous metastases are relatively frequent in RCCs and involve mainly lung, liver, and bone, and pathogenic variants in the *VHL* gene are considered the most frequent causes of hereditary and sporadic RCCs (34).

Adrenal PCCs occur in 16–20% of patients with VHLS, are usually benign, uni or bilateral, and occasionally may be multifocal, although they are malignant in 5% of cases (34). In VHLS, PCCs manifest in younger patients when compared to sporadic PCCs, and of all the PCCs diagnosed in the population, 20% of them occur in patients with VHLS (1, 7). Men with VHLS may develop papillary cystadenoma of the epididymis in 25 to 60% of cases, usually occurs during adolescence and is usually benign, multiple, and bilateral, and may be associated with male infertility (1, 7).

The involvement of the optic nerve by a HB is extremely rare and the incidence is estimated as 1.3% in VHLS (3). The optic nerve HB may grow very slowly over a number of years (35, 36). The differential diagnosis for a solitary optic nerve tumor includes an optic nerve sheath meningioma, an optic schwannoma and an optic glioma (3). Most authors recommend close radiological and ophthalmological follow-up after the diagnosis of an optic nerve HB is made and would consider surgery based on the visual progression and growth in the size of the lesion (3, 4). VHLS patients who present loss of vision not consistent with a retinal examination should be evaluated for an optic nerve HB (4). In the patient of this report, due to the slight decreased of the visual acuity in the left eye due to the optic nerve HB, it was decided only by clinical follow-up of the visual disturbances.

Detection of mosaic pathogenic variants may represent a difficulty in the diagnosis of VHLS. Mosaicism is an under-recognized phenomenon in VHLS, which could result in an overestimation of true cases of *de novo* variants. Generally, patients with mosaicism tend to present a milder clinical picture or even be asymptomatic (37). In this situation, a genetic blood test should be performed when there is clinical suspicion of VHLS or in the existence of a relative with VHLS (38, 39). Duplication and deletion research is currently performed by the multiplex ligation-dependent probe amplification (MPLA) test, and the gene sequencing includes the exons and the intron-exon junctions. When screening fails to identify a pathogenic variant in peripheral blood, screening for other tissues may be necessary for confirmation of clinical diagnosis (2).

Clinical diagnosis of the disease is made by the family history of VHLS and the presence of at least one of the disease-associated tumors (CNS and/or retinal HBs, CCRs, pancreatic neuroendocrine tumors, or papillary cystadenoma of the epididymis) (5, 6, 34). In patients without a family history, the presence of two or more HBs in the CNS or the retina or the presence of an HB in the CNS or retina and a renal or pancreatic tumor, with the exception of the cysts of the epididymis and renal cysts, both common in the general population, are indicative of VHLS (1, 5, 7).

In VHLS, the average life expectancy for men (59.4 years) is significantly higher than that for women (48.4 years) (6, 40). The mean age of death in VHLS is 49 years and is usually due to complications related to the presence of cerebellar HB or metastatic RCC (6, 40, 41).

Genetic counseling for VHLS patients should clarify the risk of recurrence of the disease in the family, the need for periodic testing of family members with the *VHL* gene mutation, treatment of disease manifestations, risk of late diagnosis, the possibility of prenatal diagnosis, and the options for the prevention of transmission of the mutated gene to descendants (1, 2). The patient in this study already has a child and a grandchild, both of whom are undergoing genetic counseling and were advised to take the genetic test for the diagnosis of VHLS, but they refused.

The early diagnosis of VHLS leads to clinical and/or surgical treatment under more favorable conditions, reducing the risks of RCC metastasis, blindness in patients with retinal HB or HB of the optical nerve, and bleeding of CNS HB, and contributes to reduction in morbidity rates and mortality related to this syndrome. VHLS patients who present with loss of vision not consistent with a retinal examination should be evaluated for an optic nerve HB.

## Ethics Statement

The studies involving human participants were reviewed and approved by Research Ethics Committee of the Faculdade de Medicina do ABC (CAEE 92678718.1.0000.0082). The patients/participants provided their written informed consent to participate in this study. Written informed consent was obtained from the individual(s) for the publication of any potentially identifiable images or data included in this article.

## Author Contributions

SB and JW conceived the study. SB, BB, and JW designed the research. SB, PC, DP, RB, and AG clinically and surgically analyzed the patient. All authors analyzed the data. SB, BB, and JW wrote the manuscript. All authors read and approved the final version of the manuscript.

### Conflict of Interest

The authors declare that the research was conducted in the absence of any commercial or financial relationships that could be construed as a potential conflict of interest.

## References

[1] Chittiboina P Lonser RR. Von Hippel–Lindau disease. Handb Clin Neurol. (2015) 132:139–56. 10.1016/B978-0-444-62702-5.00010-X 26564077 PMC5121930

[2] Ning X Zhang N Li T Wu P Wang X Li X. Telomere shortening is associated with genetic anticipation in Chinese Von Hippel–Lindau Disease Families. Cancer Res. (2014) 74:3802–9. 10.1158/0008-5472.CAN-14-0024 24986515

[3] Kanno H Osano S Shinonaga M. VHL-associated optic nerve hemangioblastoma treated with stereotactic radiosurgery. J Kidney Cancer VHL. (2018) 5:1–6. 10.15586/jkcvhl.2018.104 29911000 PMC5989481

[4] Turel MK Kucharczyk W Gentili F. Optic nerve hemangioblastomas? a review of visual outcomes. Turk Neurosurg. (2017) 27:827–31. 10.5137/1019-5149.JTN.16680-15.1 27509455

[5] Varshney N Kebede AA Owusu-Dapaah H Lather J Kaushik M Bhullar JS. A review of Von Hippel-Lindau Syndrome. J Kidney Cancer VHL. (2017) 4:20–9. 10.15586/jkcvhl.2017.88 28785532 PMC5541202

[6] Poulsen MLM Budtz-Jorgensen E Bisgaard ML. Surveillance in von Hippel-Lindau disease (vHL). Clin Genet. (2010) 77:49–59. 10.1111/j.1399-0004.2009.01281.x 19863552

[7] Shanbhogue KP Hoch M Fatterpaker G Chandarana H. von Hippel-Lindau Disease. Radiol Clin North Am. (2016) 54:409–22. 10.1016/j.rcl.2015.12.004 27153780

[8] Riley DS Barber MS Kienle GS Aronson JK von Schoen-Angerer T Tugwell P. CARE guidelines for case reports: explanation and elaboration document. J Clin Epidemiol. (2017) 89:218–35. 10.1016/j.jclinepi.2017.04.026 28529185

[9] NM_000551.3(VHL). ClinVar. (2018). Available online at: https://www.ncbi.nlm.nih.gov/clinvar/variation/182978/ (accessed September 18, 2018).

[10] Stenson PD Ball EV Mort M Phillips AD Shiel JA Thomas NST. Human Gene Mutation Database (HGMD): 2003 update. Hum Mutat. (2003) 21:577–81. 10.1002/humu.10212 12754702

[11] ACMG Board of Directors Genome editing in clinical genetics: points to consider—a statement of the American College of Medical Genetics and Genomics. Genet Med. (2017) 19:723–4. 10.1038/gim.2016.195 28125080

[12] Tabaro F Minervini G Sundus F Quaglia F Leonardi E Piovesan D. VHLdb: a database of von Hippel-Lindau protein interactors and mutations. Sci Rep. (2016) 6:31128. 10.1038/srep31128 27511743 PMC4980628

[13] Gallou C Joly D Méjean A Staroz F Martin N Tarlet G. Mutations of the VHL gene in sporadic renal cell carcinoma: definition of a risk factor for VHL patients to develop an RCC. Hum Mutat. (1999) 13:464–75. 10408776 10.1002/(SICI)1098-1004(1999)13:6<464::AID-HUMU6>3.0.CO;2-A

[14] Gallou C Longuemaux S Delomenie C Mejean A Martin N Martinet S. Association of GSTT1 non-null and NAT1 slow/rapid genotypes with von Hippel-Lindau tumour suppressor gene transversions in sporadic renal cell carcinoma. Pharmacogenetics. (2001) 11:521–35. 10.1097/00008571-200108000-00007 11505222

[15] Mattocks C Tarpey P Bobrow M Whittaker J. Comparative sequence analysis (CSA): a new sequence-based method for the identification and characterization of mutations in DNA. Hum Mutat. (2000) 16:437–43. 10.1002/1098-1004(200011)16:5<437::AID-HUMU9>3.0.CO;2-Q 11058902

[16] Iliopoulos O Ohh M Kaelin WG pVHL(19) is a biologically active product of the von Hippel–Lindau gene arising from internal translation initiation. Proc Natl Acad Sci USA. (1998) 95:11661–6. 10.1073/pnas.95.20.11661 9751722 PMC21697

[17] Iliopoulos O Kibel A Gray S Kaelin WG. Tumour suppression by the human von Hippel-Lindau gene product. Nat Med. (1995) 1:822–6. 10.1038/nm0895-822 7585187

[18] Richards FM Payne SJ Zbar B Affara NA Ferguson-Smith MA Maher ER. Molecular analysis of de novo germline mutations in the von Hippel-Lindau disease gene. Hum Mol Genet. (1995) 4:2139–43. 10.1093/hmg/4.11.2139 8589692

[19] Firth JD Ebert BL Pugh CW Ratcliffe PJ. Oxygen-regulated control elements in the phosphoglycerate kinase 1 and lactate dehydrogenase A genes: similarities with the erythropoietin 3' enhancer. Proc Natl Acad Sci USA. (1994) 91:6496–500. 10.1073/pnas.91.14.6496 8022811 PMC44229

[20] Semenza GL Roth PH Fang HM Wang GL. Transcriptional regulation of genes encoding glycolytic enzymes by hypoxia-inducible factor 1. J Biol Chem. (1994) 269:23757–63. 8089148

[21] Ebert BL Firth JD Ratcliffe PJ. Hypoxia and mitochondrial inhibitors regulate expression of glucose transporter-1 via distinct Cis-acting sequences. J Biol Chem. (1995) 270:29083–9. 10.1074/jbc.270.49.29083 7493931

[22] Semenza GL Jiang BH Leung SW Passantino R Concordet JP Maire P. Hypoxia response elements in the aldolase A, enolase 1, and lactate dehydrogenase A gene promoters contain essential binding sites for hypoxia-inducible factor 1. J Biol Chem. (1996) 271:32529–37. 10.1074/jbc.271.51.32529 8955077

[23] Kim E Zschiedrich S. Renal cell carcinoma in von Hippel-Lindau Disease-from tumor genetics to novel therapeutic strategies. Front Pediatr. (2018) 6:16. 10.3389/fped.2018.00016 29479523 PMC5811471

[24] Maxwell PH Wiesener MS Chang GW Clifford SC Vaux EC Cockman ME. The tumour suppressor protein VHL targets hypoxia-inducible factors for oxygen-dependent proteolysis. Nature. (1999) 399:271–5. 10.1038/20459 10353251

[25] Gossage L Eisen T Maher ER. VHL, the story of a tumour suppressor gene. Nat Rev Cancer. (2015) 15:55–64. 10.1038/nrc3844 25533676

[26] Kaelin WG. Cancer and altered metabolism: potential importance of hypoxia-inducible factor and 2-oxoglutarate-dependent dioxygenases. Cold Spring Harb Symp Quant Biol. (2011) 76:335–45. 10.1101/sqb.2011.76.010975 22089927 PMC4197849

[27] Niu X Zhang T Liao L Zhou L Lindner DJ Zhou M. The von Hippel-Lindau tumor suppressor protein regulates gene expression and tumor growth through histone demethylase JARID1C. Oncogene. (2012) 31:776–86. 10.1038/onc.2011.266 21725364 PMC4238297

[28] Semenza GL. Cancer-stromal cell interactions mediated by hypoxia-inducible factors promote angiogenesis, lymphangiogenesis, and metastasis. Oncogene. (2013) 32:4057–63. 10.1038/onc.2012.578 23222717 PMC4415159

[29] Semenza GL. The hypoxic tumor microenvironment: a driving force for breast cancer progression. Biochim Biophys Acta. (2016) 1863:382–91. 10.1016/j.bbamcr.2015.05.036 26079100 PMC4678039

[30] Fagerberg L Hallstrom BM Oksvold P Kampf C Djureinovic D Odeberg J. Analysis of the human tissue-specific expression by genome-wide integration of transcriptomics and antibody-based proteomics. Mol Cell Proteomics. (2014) 13:397–406. 10.1074/mcp.M113.035600 24309898 PMC3916642

[31] Ganeshan D Menias CO Pickhardt PJ Sandrasegaran K Lubner MG Ramalingam P Tumors in von Hippel-Lindau Syndrome: from head to toe-comprehensive state-of-the-art review. Radiographics. (2018) 38:849–66. 10.1148/rg.2018170156 29601266

[32] Nordstrom-O'Brien M van der Luijt RB van Rooijen E van den Ouweland AM Majoor-Krakauer DF Lolkema MP. Genetic analysis of von Hippel-Lindau disease. Hum Mutat. (2010) 31:521–37. 10.1002/humu.21219 20151405

[33] Institute of Medical Genetics in Cardiff VHL. The Human Gene Mutation Database. Available online at: http://www.hgmd.cf.ac.uk/ac/gene.php?gene=VHL (accessed September 18, 2018).

[34] Ashouri K Mohseni S Tourtelot J Sharma P Spiess PE. Implications of Von Hippel-Lindau syndrome and renal cell carcinoma. J Kidney Cancer VHL. (2015) 2:163–73. 10.15586/jkcvhl.2015.41 28326271 PMC5345519

[35] Chan C-C Chew EY Shen D Hackett J Zhuang Z. Expression of stem cells markers in ocular hemangioblastoma associated with von Hippel-Lindau (VHL) disease. Mol Vis. (2005) 11:697–704. 16163267 PMC1876780

[36] Chan C-C Collins ABD Chew EY. Molecular pathology of eyes with von Hippel-Lindau (VHL) Disease: a review. Retina. (2007) 27:1–7. 10.1097/01.iae.0000244659.62202.ee 17218907 PMC1971131

[37] Fishbein L Merrill S Fraker DL Cohen DL Nathanson KL. Inherited mutations in pheochromocytoma and paraganglioma: why all patients should be offered genetic testing. Ann Surg Oncol. (2013) 20:1444–50. 10.1245/s10434-013-2942-5 23512077 PMC4291281

[38] Sgambati MT Stolle C Choyke PL Walther MM Zbar B Linehan WM. Mosaicism in von Hippel-Lindau disease: lessons from kindreds with germline mutations identified in offspring with mosaic parents. Am J Hum Genet. (2000) 66:84–91. 10.1086/302726 10631138 PMC1288351

[39] Wu P Zhang N Wang X Li T Ning X Bu D. Mosaicism in von Hippel-Lindau disease with severe renal manifestations. Clin Genet. (2013) 84:581–4. 10.1111/cge.12092 23384228

[40] Wilding A Ingham SL Lalloo F Clancy T Huson SM Moran A. Life expectancy in hereditary cancer predisposing diseases: an observational study. J Med Genet. (2012) 49:264–9. 10.1136/jmedgenet-2011-100562 22362873

[41] Wong M Chu Y-H Tan HL Bessho H Ngeow J Tang T. Clinical and molecular characteristics of East Asian patients with von Hippel–Lindau syndrome. Chin J Cancer. (2016) 35:79. 10.1186/s40880-016-0141-z 27527340 PMC4986176

